# The pro- and anti-cancer effects of oxycodone are associated with epithelial growth factor receptor level in cancer cells

**DOI:** 10.1042/BSR20193524

**Published:** 2020-02-07

**Authors:** Yuqin Yu, Dapeng Li, Ji’an Duan, Hongshuang Xu, Li Li, Dengwu Tan, Hong Yan

**Affiliations:** 1 Department of Anesthesiology, Wuhan Central Hospital, Wuhan, Hubei Province, China; 2 Department of Anesthesiology, China Resources and WISCO General Hospital, Wuhan, Hubei Province, China; 3 Department of Respiratory Medicine, Wuhan Puren Hospital, Wuhan, Hubei Province, China; 4 Department of Anesthesiology, Lichuan People’s Hospital, Lichuan, Hubei Province, China

**Keywords:** cancer, EGFR, opioids receptor, oxidative stress, oxycodone

## Abstract

**Background:** Oxycodone is an opioid medication used for the treatment of pain in cancer patients. However, little is known on the direct effects of oxycodone on cancer cells. **Aim:** To determine the effects and mechanisms of oxycodone in cancer cells. **Materials and Methods:** Proliferation, survival and migration assays were performed on multiple types of cancer cells. Epithelial growth factor receptor (EGFR)/ERK/Akt pathway and oxidative stress were investigated after oxycodone treatment. **Results:** Oxycodone can either stimulate growth and migration without affecting survival in MDA-468 cells or inhibit growth and survival without affecting migration in SKBR3 and Caco2 cells. In addition, oxycodone can either attenuate or stimulate efficacy of chemotherapeutic drugs in cancer, depending on the type of cancer cells and nature of action of oxycodone as single drug alone. Our mechanism studies suggest that the stimulatory and inhibitory effects of oxycodone are associated with EGFR expression levels in cancer cells. In cancer cells with high EGFR level, oxycodone activates EGFR signaling in cancer cells, leading to stimulatory effects in multiple biological activities, and this is dependent on opioid receptor. In cancer cells with low EGFR level, oxycodone induces mitochondria-mediated caspase activity and oxidative stress and damage, leading to cell death. **Conclusions:** Our work is the first to demonstrate systematic analysis of oxycodone’s effects and mechanism of action in cancer. The activation of EGFR signaling by oxycodone may provide a new guide in the clinical use of oxycodone, in particular for cancer patients with high EGFR levels.

## Introduction

Opioids are substances that acts on μ-, κ- and δ-opioid receptors, which are mainly found in the neuronal system and the gastrointestinal tract [1]. Although opioid abuse is a health care crisis globally, opioids remain the cornerstone of pain management in cancer [2]. Opioids are often administrated concurrently with anticancer drugs to patients with advanced disease and exhibit potent analgesic effects without excessive adverse effects [3]. Apart from their analgesic effects, recent evidence suggest that opioids might influence cancer progression via mediating angiogenesis and/or biological activities of tumor cells [4]. Among the clinically used opioids, such as morphine, oxycodone and fentanyl, the role of morphine in cancer biology has been studied the most. Although the effects of morphine on tumor growth are still inconclusive, the majority of studies demonstrated effects of morphine on the growth, migration and survival of cancer cells. It enhanced renal cell carcinoma aggressiveness and promoted breast cancer stem cell properties that contributed to breast cancer progression and chemoresistance [5–7].

Oxycodone is a semi-synthetic opioid agent and the equivalent analgesic dose of oxycodone is two-thirds of morphine [8]. Although the affinity of oxycodone for the μ-opioid receptor is lower than that of morphine, oxycodone fully activates the κ-opioid receptor that can produce an analgesic effect without causing euphoria, gastrointestinal motility inhibition and respiratory inhibition [9,10]. However, the effects of oxycodone on cancer cells have not been revealed. Only one study by Tian et al. demonstrated that oxycodone inhibits proliferation and migration, and induces apoptosis in human lung adenocarcinoma cells, with a similar potency as that of morphine [11]. The aim of the present study was to determine the effects and underlying mechanisms of oxycodone in cancer using breast and colon cancer cells with varying histological, cellular and genetic origins.

## Materials and methods

### Drugs, compounds, antibodies and cell culture

Minimal Essential Media (MEM), Penicillin-Streptomycin (10,000 U/ml) and fetal bovine serum (FBS) were purchased from Thermo Fisher Scientific Inc., U.S.A. Oxycodone hydrochloride tablet (Sankyo Pharmaceutical Co. Ltd., Japan) was obtained from the Department of Pharmacy, Wuhan Central Hospital. Benzyloxycarbonyl-Val-Ala-Asp (OMe) fluoromethylketone (Z-VAD-fmk), α-tocopherol and N-acetyl cysteine (NAC) were purchased from Sigma, U.S.A. All antibodies were purchased from Santa Cruz Biotechnology Inc., U.S.A. MDA-468, SKBR and Caco2 (KeyGen Biotech Co., Ltd. China) were grown in 75 cm^2^ culture flask using MEM supplemented with final concentration of 10% FBS and 100 U/ml Penicillin–Streptomycin under 37°C, 100% air and humid atmosphere.

### BrdU proliferation

Cells were plated onto 96-well plate. When reaching 70% confluency, drugs were added to the well. After 3 days treatment, bromodeoxyuridine/5-bromo-2′-deoxyuridine (BrdU) was added to the wells and incorporated into the DNA of dividing cells. Proliferation level was measured using BrdU Cell Proliferation Assay Kit (Abcam, U.S.A.).

### Apoptosis assay

Cells were plated onto 96-well plate. When reaching 70% confluency, drugs were added to the well. After 3 days treatment, cells were harvested using trypsin. Apoptotic cells were stained with FITC Annexin V Apoptosis Detection Kit (BioLegend, U.S.A.) and quantified using flow cytometry.

### Boyden chamber migration assay

Migration assay was assessed after 8 h of exposure to drugs using the Boyden chamber assay as previously described [12]. A total of 10,000 cells together with drugs in 200 μl MEM medium supplemented with 2% FBS were placed into the Falcon cell culture insert with 8-µm pore size polycarbonate membrane filters (Corning Inc., U.S.A.). The lower chamber was filled with 600 μl MEM medium supplemented with 10% FBS. After incubation for 8 h in tissue incubator, the cells on the upper side of the inserts were scrapped off. Migrated cells on the lower surface of the inserts were fixed with 10% formalin and stained with 0.4% Crystal Violet (Sigma, U.S.A.). Cells from five independent visual fields were counted under the microscope.

### Denaturing sodium dodecyl sulfate–polyacrylamide gel electrophoresis (SDS–PAGE) and Western blot (WB) analyses

After 24 h drug treatment, total proteins were extracted from cells with RIPA lysis buffer and measured using the bicinchoninic acid protein assay kit (Biyuantian Inc., China). Protein lysates were separated by denaturing SDS-PAGE and analyzed by WB with designated primary and secondary antibodies as per standard protocol. Signals were detected using the ECL Plus Kit (Biyuntian Inc., China).

### Active caspase activity assays

Active caspase activity was assessed after 24 h of exposure to drugs using Caspase-3, -8 and -9 Assay Kits (Abcam Inc., U.S.A.) according to manufacturer’s protocols. These assays were based on spectrophotometric detection of the chromophore ρ-nitroaniline (ρNA) after it is cleaved from IETD-ρNA (for active caspase 8), DEVD-ρNA (for active caspase 3) and LEHD-ρNA (for active caspase 9).

### Oxidative stress and DNA damage assays

Intracellular reactive oxygen species (ROS) level (an indicator of oxidative stress) and 8-hydroxy-2′-deoxyguanosine (8-OHdG, an indicator of oxidative DNA damage [13]) were assessed after 24 h drug treatment. ROS was measured using ROS Detection Assay Kit (Biovision Inc., U.S.A.). 8-OHdG levels were quantified using the OxiSelect Oxidative DNA Damage ELISA Kit (Cell Biolabs Inc., U.S.A.), following the manufacturer’s protocol. Absorbance at 450 nm was detected on a Spectramax M5 Microplate Reader.

### Statistical analysis

All data were expressed as mean ± SD. To compare values between multiple groups, analysis of variance (ANOVA) was applied. Statistical significance was defined as *P* less than 0.05.

## Results

### The differential effects of oxycodone are cancer cell type-dependent

**Figure 1:**
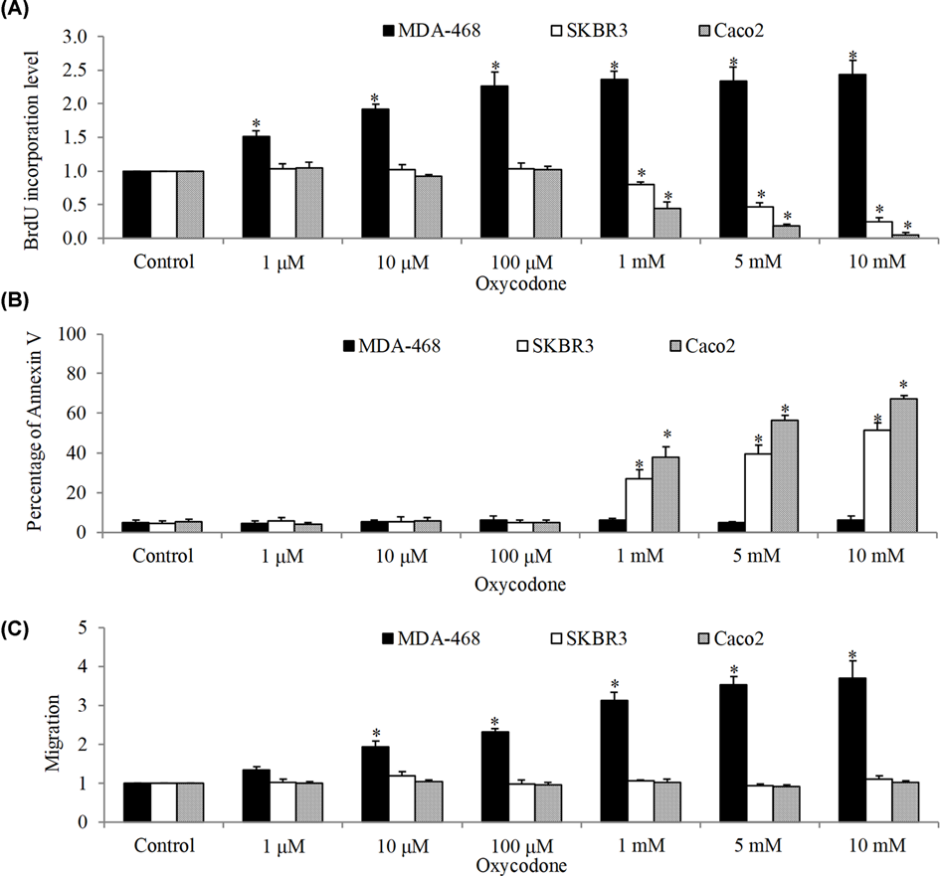
The effects of oxycodone are cancer cell type-dependent The differential effects of oxycodone on growth (**A**), survival (**B**) and migration (**C**) in breast cancer (MDA-468 and SKBR3) and colon cancer (Caco2) cells. Oxycodone at concentration ranging from 0.01 to 10 mM were tested. *, *P* < 0.05, compared with control.

The effects of oxycodone on several types of cancer cell growth, survival and migration were examined. We found that oxycodone starting from 1 μM significantly increased proliferation of MDA-468 in a dose-dependent manner as assessed by measuring incorporation of BrdU (Figure 1A). We observed the increasing trend up to 100 μM and it reached saturation thereafter. Interestingly, oxycodone did not influence growth of SKBR3 and Caco2 cells up to 100 μM. In contrast, oxycodone at 1–10 mM dose-dependently decreased proliferation of SKBR3 and Caco2 cells. In addition, oxycodone did not affect MDA-468 cell survival up to 10 mM whereas significantly induced apoptosis in SKBR3 and Caco2 (Figure 1B). Oxycodone stimulated migration of MDA-468 cells but did not affect the migration of SKBR3 and Caco2 cells (Figure 1C). Similar to its effects on MDA-468 cell growth, the stimulatory effect of oxycodone on migration reached saturation at 100 μM. These results demonstrate that oxycodone has differential effects on cancer cell activities, depending on cancer cell type.

### The differential combinatory effects of oxycodone with chemotherapeutic drugs are cancer cell type-dependent

**Figure 2:**
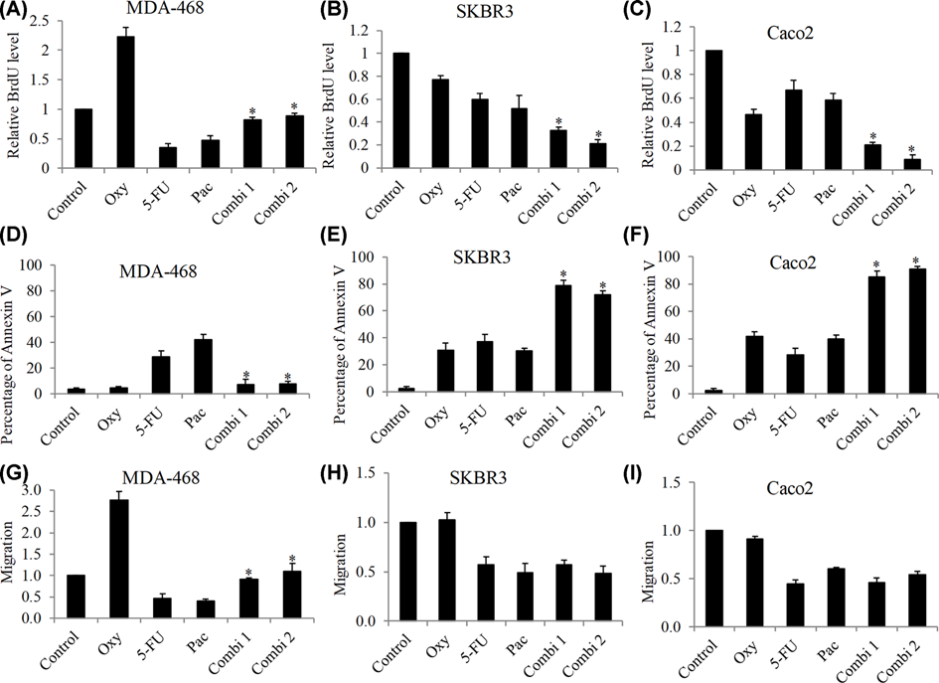
The differential combinatory effects of oxycodone with chemotherapeutic drugs in cancer cells Oxycodone (1 mM) significantly alleviated paclitaxel and 5-FU’s inhibitory effects on growth (**A**), survival (**D**) and (**G**) migration in MDA-468 cells. Oxycodone (1 mM) significantly enhanced paclitaxel and 5-FU’s inhibitory effects on growth (**B,C**) and survival (**E,F**) in SKBR3 and Caco2 cells. Oxycodone (1 mM) did not affect paclitaxel and 5-FU’s inhibitory effects on migration (**H,I**) in SKBR3 and Caco2 cells. About 50 nM of paclitaxel and 100 nM of 5-FU were used in combination studies. Combi1 is the combination of oxycodone and paclitaxel. Combi2 is the combination of oxycodone and 5-FU. *, *P* < 0.05, compared with paclitaxel or 5-FU.

We next investigated whether oxycodone interferes with the inhibitory effects of chemotherapeutic drugs in these cancer cells. Paclitaxel and 5-FU are commonly used for chemotherapy in many solid cancers. We found that oxycodone at concentration that promotes growth as single drug alone significantly alleviated the anti-proliferative effect of both 5-FU and paclitaxel in MDA-468 cells (Figure 2A). Oxycodone at concentration that inhibits growth as single drug alone significantly enhanced the anti-proliferative effect of both 5-FU and paclitaxel in SKBR3 and Caco2 cells (Figure 2B,C). Notably, oxycodone at concentration that does not affect apoptosis as single drug alone also significantly alleviated pro-apoptotic effect of chemotherapeutic drugs in MDA-468 cells (Figure 2D). The combination was significantly more effective in inducing apoptosis than chemo drugs in SKBR3 and Caco2 cells (Figure 2E,F). In addition, oxycodone alleviated the anti-migratory effects of chemotherapeutic drugs in MDA-468, whereas it did not affect their anti-migratory effects in SKBR3 and Caco2 cells (Figure 2G–I). These results indicate that oxycodone can either attenuate or enhance the efficacy of chemotherapy, depending on cancer cell type.

### Oxycodone activates EGFR signaling in MDA-468 but not SKBR3 or Caco2 cells

**Figure 3:**
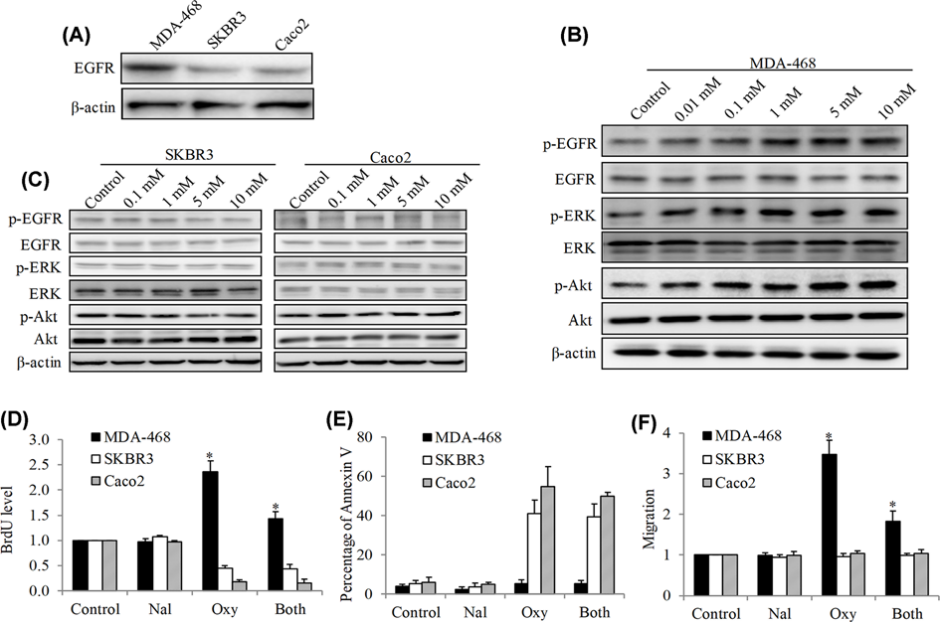
Oxycodone activates EGFR signaling in MDA-468 but not SKBR3 or Caco2 cells (**A**) Representative Western blot (WB) photo showing EGFR level in MDA-468, SKBR3 and Caco2 cells. Representative WB phot showing p-EGFR, p-ERK and p-Akt levels in MDA-468 (**B**), SKBR3 and Caco2 (**C**) cells after 24 h treatment of oxycodone. Naloxone (500 nM) significantly reversed the effects of oxycodone (5 mM) on growth (**D**), survival (**E**) and migration (**F**) in MDA-468 but not SKBR3 or Caco2 cells. *P < 0.05, compared with oxycodone.

Compared with SKBR3 and Caco2, our mechanism analysis indicated that MDA-468 displayed higher expression level (Figure 3A). We next analyzed the EGFR downstream signaling using Western immunoblotting approach in MDA-468, SKBR3 and Caco2 cells after 24 h of oxycodone treatment. We found that oxycodone increased the phosphorylation of EGFR at Tyr1173, phosphorylation of ERK at Thr202/Tyr204 and phosphorylation of Akt at Thr308 in MDA-468 cells (Figure 3B), suggesting that oxycodone activates EGFR signaling in MDA-468 cells. Notably, the concentrations of oxycodone that activated EGFR are consistent with the ones that activates growth and migration. In contrast, oxycodone did not affect phosphorylation level of EGFR, ERK and Akt in SKBR3 and Caco2 cells (Figure 3C).

Naloxone serves as a competitive receptor antagonist with binding affinity to μ, δ and κ opioid receptors [14]. We found that naloxone significantly inhibited oxycodone-induced stimulation of growth and migration in MDA-468 cells without affecting oxycodone-induced inhibition of growth and survival in SKBR3 and Caco2 cells (Figure 3D–F). Taken together, these results demonstrate that oxycodone activates EGFR to stimulate growth and migration via opioid-receptor-dependent manner only in MDA-468 cells. In addition, other mechanisms are involved in oxycodone’s action on SKBR3 and Caco2 cells.

### Oxycodone increases caspase-dependent apoptosis via inducing oxidative stress and damage in SKBR3 and Caco2 but not MDA-468 cells

**Figure 4:**
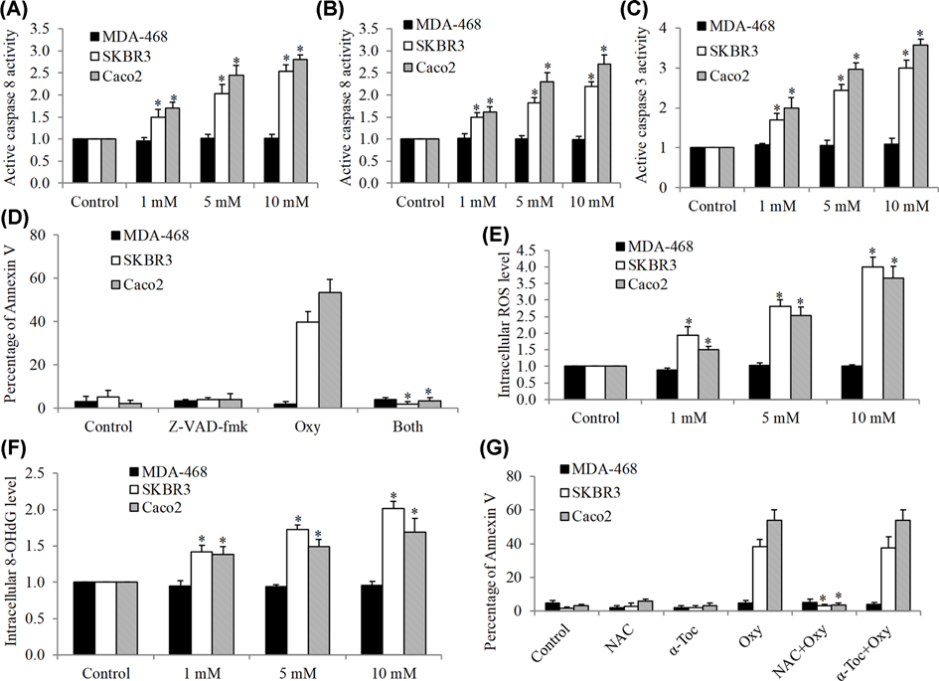
Oxycodone induces caspase-dependent apoptosis, oxidative stress and damage in SKBR3 and Caco2 but not MDA-468 cells Oxycodone significantly increased active caspase 8 (**A**), active caspase 9 (**B**) and active caspase 3 (**C**) activities in SKBR3 and Caco2 but not MDA-468 cells. (**D**) The pro-apoptotic effect of oxycodone (5 mM) was abolished in the presence of Z-VAD-fmk (50 μM) in SKBR3 and Caco2 cells. Both are Z-VAD-fmk and oxycodone. Oxycodone significantly increased intracellular ROS (**E**) and 8-OHdG (**F**) in SKBR3 and Caco2 cells but not MDA-468 cells. (**G**) NAC but not α-tocopherol significantly reversed the pro-apoptotic effect of oxycodone in SKBR3 and Caco2 cells. Antioxidants α-tocopherol and NAC were used at 10mM. *P < 0.05, compared with control or oxycodone.

We next determined the underlying mechanisms of oxycodone in inducing apoptosis in SKBR3 and Caco2 cells. We used MDA-468 cells as the negative control. We found that oxycodone dose-dependently increased active caspase 8, 9 and 3 levels in SKBR3 and Caco2 but not MDA-468 cells (Figure 4A–C). In addition, the concentrations of oxycodone that activate caspase activities are the same with the ones that induce apoptosis. In line with expectations, a pan-caspase inhibitor Z-VAD-fmk completely reversed the pro-apoptotic effects of oxycodone in SKBR3 and Caco2 cells (Figure 4D). We further found that oxycodone increased intracellular ROS and 8-OHdG levels (Figure 4E,F), suggesting the induction of oxidative stress and damage in SKBR3 and Caco2 cells. N-acetyl-L-cysteine (NAC), an antioxidant that inhibits mitochondrial thiol redox-mediated ROS production, but not α-tocopherol, an antioxidant that inhibits lysosomal lipid-mediated ROS production, protects against oxycodone-induced apoptosis (Figure 4G), suggesting that oxycodone induces caspase activity, oxidative stress and apoptosis via mitochondrial pathway in SKBR3 and Caco2 cells.

## Discussion

Although opioids have been considered as routine medicine for pain management of cancer patients, in particular those with metastasis, the effects of opioids on cancer progression, metastasis and recurrence are increasingly being questioned. There is evidence that opioids can affect multiple processes that critically contributes to cancer progression, including immune system function, angiogenesis, apoptosis and invasion, yet no available clinical data support that opioids promote cancer progression [15]. In addition, most preclinical studies on the effects of opioids in cancer have involved morphine that demonstrates both growth-promoting and growth-inhibiting effects in cancer cells [16]. Nevertheless, evidence for the effects of opioids on cancer is mixed and inconclusive [17,18]. The present study evaluated the effects of oxycodone on breast and colon cancer cells, and demonstrated that oxycodone has differential effects in cancer, depending on the type of cancer cells.

Little is known on the effects of oxycodone on cancer. This work provides proof of concept and indications to demonstrate the differential effects of oxycodone on cancer cells. To achieve the objectives, we therefore performed a systematic analysis by testing the effects over a wide range (10,000-fold change) of concentrations (from 1 μM to 10 mM) of oxycodone in various types of cancer cells: two breast cancer cell lines (e.g. MDA-468 and SKBR3) and one colon cancer cell line (e.g. Caco2). We found that oxycodone can either stimulate or inhibit cancer cell growth (Figure 1A). This is less likely due to the differences of drug concentrations or cancer type because both pro- and anti-proliferative effects of oxycodone were observed on the same concentration range (1, 5 and 10 mM) in both breast cancer (SKBR3) and colon cancer (Caco2) cells (Figure 1A). In addition, oxycodone only induced apoptosis and increased migration in some cell lines (Figure 1B,C), suggesting that apoptosis induction and migration stimulation might not be the persistent action of oxycodone in cancer cells. The studies on oxycodone’s activity in cancer are limited. One report showed that oxycodone at high micromolar concentration inhibited migration and proliferation, and induced apoptosis in A549 lung cancer cells [11]. Another showed that oxycodone at millimolar concentration decreased viability in SH-SY5Y neuroblastoma cells [19]. Our work is consistent with the previous work on the anti-cancer activity of oxycodone, and is the first to show that oxycodone has pro-cancer activity. About 0.5 μg/ml (∼1.6 μM) of oxycodone in plasma was detected in patients after receiving 160 mg/day [20]. In addition, 8 μg/ml (∼25 μM) of oxycodone was detected in cerebrospinal fluid after epidural oxycodone administration [21]. These suggest that concentrations of oxycodone we had tested in our study are clinically relevant. Interestingly, this feature of oxycodone has also been observed in morphine [5,22], suggesting that opioids have both pro- and anti-cancer activity. In addition, we further found that oxycodone can either attenuate or augment efficacy of chemotherapeutic drugs, depending on the type of cancer cells and nature of action of oxycodone as single drug alone (Figure 2).

A significant finding of our work is that the stimulatory and inhibitory effects of oxycodone are associated with EGFR expression level in cancer cells (Figures 3 and 4). We found that MDA-468 had much higher level in EGFR than SKBR3 and Caco2 (Figure 3A). Oxycodone activates EGFR signaling in MDA-468 but not SKBR3 or Caco2 cells (Figure 3B,C). Opioid receptor antagonist naloxone significantly reversed the stimulatory effects of oxycodone in MDA-468 without affecting the inhibitory effects of oxycodone in SKBR3 and Caco2 cells (Figure 3D–F), demonstrating that the capability of oxycodone in activating EGFR and stimulating MDA-468 cells is dependent on the opioid receptor. This is consistent with Nanomi et al’s observations that morphine induces EGFR activation in H2009 lung cancer cells due to the co-localization of EGFR and μ-opioid receptor [23].

In addition, in cancer cells with low EGFR expressions, oxycodone induces mitochondria-mediated caspase activity, oxidative stress and damage, leading to cancer cell death (Figure 4). This is in contrast with the previous work that morphine has antioxidant, anti-apoptotic and cytoprotective properties in cardiomyocytes [24]. The reason behind this discrepancy is likely to be a result of differences in drug concentrations, cell types and drugs. Morphine demonstrates both growth-promoting and growth-inhibiting effects in cancer cells [16]. Similarly, our work also showed that oxycodone has differential effects in cancer, depending on cancer type. We noted that the concentration of morphine used in Hossein et al’s work was as low as 1 μM [24], whereas the pro-apoptotic concentration of oxycodone shown in our work started from 1 mM. In addition, the cytotoxicity of oxycodone has been shown in hepatocellular carcinoma and neuroblastoma cells [25]. Our findings extend the previous work on the identification of mitochondria-mediated oxidative stress as the mechanism of oxycodone’s cytotoxicity in cancer cells.

In conclusion, our findings provide preclinical evidence to show that oxycodone displays varying effects in cancer with different underlying mechanisms, and this is associated with EGFR expression level in cancer cells.

## Abbreviations

8-OHdG: 8-hydroxy-2′-deoxyguanosine
EGFR: epithelial growth factor receptor
FBS: fetal bovine serum
MEM: Minimal Essential Media
NAC: N-acetyl-L-cysteine
ROS: reactive oxygen species
